# Supplementary material for: Vulnerabilities of the SARS-CoV-2 Virus to Proteotoxicity—Opportunity for Repurposed Chemotherapy of COVID-19 Infection
Source: Front Pharmacol. 2020 Oct 9;11:585408. doi: 10.3389/fphar.2020.585408 (PMC7581855; doi:10.3389/fphar.2020.585408)
Supplement: Supplementary Table 1 — SARS-CoV-2 viral proteome. *Sequence from reference (Chan et al., 2020). [file DataSheet_1.docx]

**SUPPLEMENTARY INFORMATION.**

**Table S1 │ SARS-CoV-2 viral proteome.**

| No | Protein (acronym(s)) | Reference sequence  (no of a.a. residues) | Function |
| --- | --- | --- | --- |
|  | Non-structural protein 1 (nsp1) | YP_009725297.1  (180 residues) | Inhibits host translation by interacting with the 40S ribosomal subunit. |
|  | Non-structural protein 2 (nsp2) | YP_009725298.1  (638 residues) | May modulate host cell survival signaling pathway by interacting with host prohibitin-1 and prohibitin-2. |
|  | Non-structural protein 3 /Papain-like proteinase (nsp3, PL-PRO) | YP_009725299.1  (1945 residues) | Cleaves N-terminus of the replicase polyprotein. With nsp4, participates in assembly of viral membrane assembly. Antagonizes type I interferon innate immunity by. Prevents host NF-kappa-B signaling. |
|  | Non-structural protein 4 (nsp4) | YP_009725300.1  (500 residues) | Participates in the virion double-membrane vesicles assembly. |
|  | Non-structural protein 4 (nsp5, 3C-like proteinase, 3CL-PRO, Main protease) | YP_009725301.1  (306 residues) | Cleaves C-terminus of replicase polyprotein R1ab at 11 sites. |
|  | Non-structural protein 6 (nsp6) | YP_009725302.1  (290 residues) | RoleRRole in initial induction and regulation of autophagosomes from host reticulum endoplasmic. |
|  | Non-structural protein 7 (nsp7) | YP_009725303.1  (83 residues) | Forms a hexadecamer with nsp8 that may participate in viral replication by acting as a primase or may synthesize longer oligonucleotides. |
|  | Non-structural protein 8 (nsp8) | YP_009725304.1  (198 residues) | Forms a complex with nsp7 that may act as a primase or may synthesize longer oligonucleotides. |
|  | Non-structural protein 9 (nsp9) | YP_009725305.1  (113 residues) | May be an ssRNA-binding protein participating in viral replication. |
|  | Non-structural protein 10 (nsp10) | YP_009725306.1  (139 residues) | Pivotal role in viral transcription by stimulating both nsp14 3'-5' exoribonuclease and nsp16 2'-O-methyltransferase activities for viral mRNAs cap methylation. |
|  | Non-structural protein 11 (nsp11) | YP_009725312.1  (13 residues) |  |

**Table S1│SARS-CoV-2 viral proteome (cont’d).**

| No | Protein (acronym(s)) | Reference sequence  (no of a.a. residues) | Function |
| --- | --- | --- | --- |
|  | RNA-directed RNA polymerase (nsp12, Pol/RdRp) | YP_009725307.1  (932 residues) | Replication and transcription of the viral RNA genome. Part of orf1ab polyprotein. |
|  | Helicase (nsp13, Hel) | YP_009725308.1  (601 residues) | Multi-functional protein with RNA and DNA duplex-unwinding activities with 5' to 3' polarity. |
|  | Guanine-N7 methyl-transferase (nsp14, ExoN) | YP_009725309.1  (527 residues) | Exoribonuclease activity acting on both ssRNA and dsRNA in a 3' to 5' direction. N^7^-guanine methyltransferase activity. |
|  | Uridylate-specific endoribonuclease (nsp15, NendoU) | YP_009725310.1  (346 residues) | Mn^2+^-dependent, uridylate-specific endoribonuclease. |
|  | 2'-O-ribose methyl-transferase (nsp16) | YP_009725311.1  (298 residues) | Methyltransferase mediating mRNA cap 2'-O-ribose methylation to the 5'-cap structure of viral mRNAs - prerequisite for binding of nsp16. Essential function to evade immune system. |
|  | Spike glycoprotein (Spike protein S1, S-glycoprotein) | YP_009724390.1  (1273 residues) | Attaches the virion to the cell membrane by interacting with host receptor, initiating the infection. Binding to human ACE2 and CLEC4M/DC-SIGNR receptors. |
|  | Protein 3a (ORF3a) | YP_009724391.1  (275 residues) | Forms K^+^-sensitive ion channels which may modulate virus release. Up-regulates fibrinogen subunit expression in host lung epithelial cells. Downregulates the type 1 interferon receptor. |
|  | Protein 3b (ORF3b)* | (57 residues) | Inhibits expression of ß-interferon. |
|  | Envelope small membrane protein (E protein) | [YP_009724392.1](https://www.ncbi.nlm.nih.gov/protein/YP_009724392.1)  (75 residues) | AViroporin in host membranes forming pentameric protein-lipid pores allowing ion transport. Also involved in the induction of apoptosis. |
|  | Membrane protein (M protein) | YP_009724393.1  (222 residues) | Component of the viral envelope involved in virus morphogenesis and assembly. |
|  | Non-structural protein 6 (ns6, ORF6) | [YP_009724394.1](https://www.ncbi.nlm.nih.gov/protein/YP_009724394.1)  (61 residues) | Determinant of virus virulence. |

**Table S1│SARS-CoV-2 viral proteome (cont’d).**

| No | Protein (acronym(s)) | Reference sequence  (no of a.a. residues) | Function |
| --- | --- | --- | --- |

|  | Protein 7a (ORF7a) | YP_009724395.1  (121 residues) | Non-structural protein of unknown function |
| --- | --- | --- | --- |
|  | Protein non-structural 7b (ORF7b) | YP_009725318.1  (43 residues) | Unknown function |
|  | Protein non-structural 8 (ORF8) | YP_009724396.1  (121 residues) | May play a role in host virus interaction. |
|  | Nucleoprotein (NC, ORF9) | YP_009724397.2  (419 residues) | Packages the positive strand viral genome RNA into a helical ribonucleocapsid. Also enhances efficiency of viral RNA transcription and viral replication. |
|  | Protein 9b (ORF9b) | ORF9B_WCPV  (97 residues) | Unknown function. |
|  | ORF10 protein | YP_009725255.1  (38 residues) | Unknown function. |
|  | Uncharacterized protein 14 (ORF14) | Y14_WCPV  (73 residues) | Unknown function. |

*Sequence from reference ([Chan et al., 2020](#_ENREF_1)).

**Table S2│Receptor binding domain of SARS-CoV-2 viral proteome.**

| Amino acid | N | | Prevalence | | Proportion in RBD (%) | Fold Enrichment |
| --- | --- | --- | --- | --- | --- | --- |
|  | All | (RBD) | All | RBD |  |  |
| Ala | 667 | 18 | 6.76 | 2.9 | 2.7 | 0.4 |
| Arg | 358 | 110 | 3.63 | 17.7 | 30.7 | 4.9 |
| Asn | 537 | 42 | 5.44 | 6.8 | 7.8 | 1.2 |
| Asp | 514 | 45 | 5.21 | 7.3 | 8.8 | 1.4 |
| Cys | 310 | 15 | 3.14 | 2.4 | 4.8 | 0.8 |
| Gln | 369 | 29 | 3.74 | 4.7 | 7.9 | 1.3 |
| Glu | 452 | 41 | 4.58 | 6.6 | 9.1 | 1.4 |
| Gly | 574 | 22 | 5.82 | 3.5 | 3.8 | 0.6 |
| His | 193 | 17 | 1.96 | 2.7 | 8.8 | 1.4 |
| Ile | 514 | 8 | 5.21 | 1.3 | 1.6 | 0.3 |
| Leu | 950 | 27 | 9.63 | 4.4 | 2.8 | 0.5 |
| Lys | 568 | 74 | 5.76 | 11.9 | 13.0 | 2.1 |
| Met | 203 | 7 | 2.06 | 1.1 | 3.5 | 0.6 |
| Phe | 504 | 13 | 5.11 | 2.1 | 2.6 | 0.4 |
| Pro | 404 | 23 | 4.09 | 3.7 | 5.7 | 0.9 |
| Ser | 665 | 53 | 6.74 | 8.5 | 8.0 | 1.3 |
| Thr | 728 | 33 | 7.38 | 5.3 | 4.5 | 0.7 |
| Trp | 113 | 2 | 1.15 | 0.3 | 1.8 | 0.3 |
| Tyr | 448 | 23 | 4.54 | 3.7 | 5.1 | 0.8 |
| Val | 796 | 18 | 8.07 | 2.9 | 2.3 | 0.4 |
| Total: | 9867 | 620 | 100 | 100 |  |  |

RBD analysis was applied to SARS-CoV-2 proteome (see Table S1) using a window of 5 amino acids and gyration angle between two consecutive residues in the sequence of 100° ([Gallet et al., 2000](#_ENREF_2)).

**REFERENCES**

Chan, J.F.-W., Kok, K.-H., Zhu, Z., Chu, H., To, K.K.-W., Yuan, S., and Yuen, K.-Y. (2020). Genomic characterization of the 2019 novel human-pathogenic coronavirus isolated from a patient with atypical pneumonia after visiting Wuhan. *Emerging Microbes & Infections* 9**,** 221-236.

Gallet, X., Charloteaux, B., Thomas, A., and Braseur, R. (2000). A fast method to predict protein interaction sites from sequences. *J.Mol.Biol.* 302**,** 917-926.
